# Oral Mesenchymal Stem/Progenitor Cells: The Immunomodulatory Masters

**DOI:** 10.1155/2020/1327405

**Published:** 2020-02-25

**Authors:** Li-li Zhou, Wei Liu, Yan-min Wu, Wei-lian Sun, C. E. Dörfer, K. M. Fawzy El-Sayed

**Affiliations:** ^1^Department of Periodontology, The Second Affiliated Hospital, School of Medicine, Zhejiang University, Hangzhou 310009, China; ^2^Key Laboratory of Oral Biomedical Research of Zhejiang Province, Zhejiang University School of Stomatology, China; ^3^Clinic for Conservative Dentistry and Periodontology, School of Dental Medicine, Christian-Albrechts-Universität zu Kiel, Kiel 24105, Germany; ^4^Oral Medicine and Periodontology Department, Faculty of Oral and Dental Medicine, Cairo University, Cairo 11435, Egypt

## Abstract

Oral mesenchymal stem/progenitor cells (MSCs) are renowned in the field of tissue engineering/regeneration for their multilineage differentiation potential and easy acquisition. These cells encompass the periodontal ligament stem/progenitor cells (PDLSCs), the dental pulp stem/progenitor cells (DPSCs), the stem/progenitor cells from human exfoliated deciduous teeth (SHED), the gingival mesenchymal stem/progenitor cells (GMSCs), the stem/progenitor cells from the apical papilla (SCAP), the dental follicle stem/progenitor cells (DFSCs), the bone marrow mesenchymal stem/progenitor cells (BM-MSCs) from the alveolar bone proper, and the human periapical cyst-mesenchymal stem cells (hPCy-MSCs). Apart from their remarkable regenerative potential, oral MSCs possess the capacity to interact with an inflammatory microenvironment. Although inflammation might affect the properties of oral MSCs, they could inversely exert a multitude of immunological actions to the local inflammatory microenvironment. The present review discusses the current understanding about the immunomodulatory role of oral MSCs both in periodontitis and systemic diseases, their “double-edged sword” uniqueness in inflammatory regulation, their affection of the immune system, and the underlying mechanisms, involving oral MSC-derived extracellular vesicles.

## 1. Introduction

According to the International Society for Cellular Therapy, mesenchymal stem/progenitor cells (MSCs) positively express the surface markers CD73, CD90, and CD105 and negatively express the endothelial as well as the hematopoietic markers CD11b, CD19, CD79*α*, CD31, CD34, CD45, and HLA-DR antigen [1]. CD90 (Thy-1) is usually used as a marker for a variety of MSCs and for the axonal processes of mature neurons. CD105 (endoglin), as a part of the TGF-*β* receptor complex that is involved in the binding of TGF-*β*1, TGF-*β*3, BMP-2, and/or BMP-7, has been found on endothelial cells, activated macrophages, fibroblasts, smooth muscle cells, and MSCs. CD73, as a marker of lymphocyte differentiation, can be coexpressed with CD90 and CD105 in very high concentrations on any potential MSCs. Thus, CD73, CD90, and CD10 are three major makers expressed on the MSCs' surface. CD79a and CD19 are expressed on B cells, and CD34 is a marker of primitive hematopoietic progenitors and endothelial cells. CD14 and CD11b are predominantly expressed on monocytes and macrophages, and CD45 is a marker of pan-leukocyte. HLA-DR molecules are not expressed on MSCs unless stimulated. Accordingly, MSCs lack the expression of CD45, CD34, CD14 or CD11b, CD79a or CD19, and HLA-DR antigens [1].

MSCs are characterized by their self-renewal and multilineage differentiation capability into osteogenic, adipogenic, chondrogenic, and myogenic- and neurogenic-like lineages. Aside from their remarkable proliferative and multilineage differentiation/regenerative potential [2], multiple impressive paracrine functions, which affect the surrounding microenvironment, have been ascribed to MSCs [3]. These paracrine activities unfolded over the last years to encompass remarkable modulatory effects in the courses of a variety of autoimmune and inflammatory diseases, including multiple sclerosis, acute lung injury, muscular dystrophy, osteoarthritis, and graft versus host disease (GVHD) [4]. The observed effects were primarily attributed to the MSCs' potential to constrain the underlying immune responses, while absconding the host's immune surveillance [5]. Yet, although we mostly consider the effects of MSCs on their surrounding environment, the aforementioned biological attributes of MSCs can be greatly influenced by their inflammatory microenvironment [6], which usually present during many diseases as well as the initial physiological healing processes.

**Figure 1:**
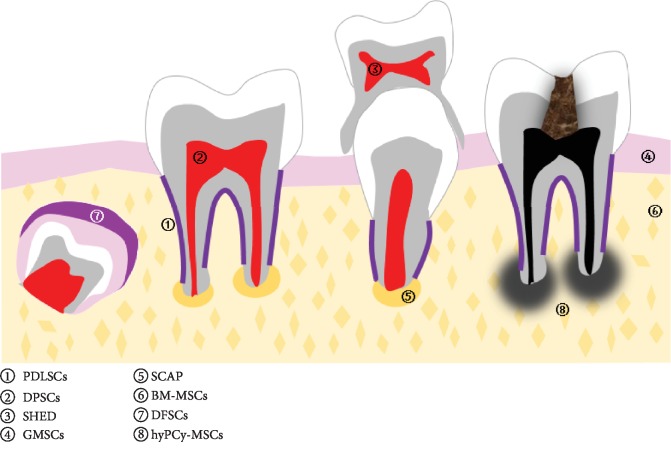
Location of oral-tissue-derived MSCs. PDLSCs: periodontal ligament stem cells; DPSCs: dental pulp stem cells; SHED: stem cells from human exfoliated deciduous teeth; GMSCs: gingival mesenchymal stem cells; SCAP: stem cells of the apical papilla; DFSCs: dental follicle stem cells; BM-MSCs: bone marrow mesenchymal stem cells; PCy-MSCs: periapical cyst-mesenchymal stem cells.

Originally, MSCs were characterized as fibroblast-like cells isolated from the bone marrow mesenchymal cellular populations [7]. Since the turn of the millennium, oral tissues have further been described as readily available sources for MSCs' isolation. Relying on their ease of acquisition and remarkable multilineage differentiation potential, oral-tissue-derived MSCs have attracted pronounced attention [8] (Figure 1). Periodontal ligament stem/progenitor cells (PDLSCs), residing inside the periodontal ligament, express embryonic stem cell markers (Oct4, Sox2, Nanog, and Klf4) and a subset of neural crest markers (Nestin, Slug, p75, and Sox10) [9]. The gingival mesenchymal stem/progenitor cells (GMSCs) contain two subpopulations of G-MSCs: 90% neural-crest-derived GMSCs (N-GMSCs) and 10% mesoderm-derived GMSCs (M-GMSCs), where N-GMSCs are more capable of differentiating into neural cells than M-GMSCs [10]. Dental pulp stem/progenitor cells (DPSCs) and stem/progenitor cells are from human exfoliated deciduous teeth (SHED) originate from dental pulps of adult as well as deciduous teeth, respectively. Stem/progenitor cells from the apical papilla (SCAP) reside in the apical papilla of incompletely developed teeth, and dental follicle stem/progenitor cells (DFSCs) could be isolated from human third molars. The alveolar bone proper further harbors bone marrow mesenchymal stem/progenitor cells (BM-MSCs). Remarkably, MSCs isolated in the course of the surgical removal of periapical cyst were termed as human periapical cyst-mesenchymal stem cells (hPCy-MSCs) [11]. Though considered as “biological waste,” hPCy-MSCs presented more potent differentiation toward neurogenesis and osteogenesis [12] and were administrated in neurodegenerative diseases like Parkinson's disease and in bone regeneration [12–14]. However, evidence of immunomodulatory properties about hPCy-MSCs is still absent [15]. Besides, oral MSCs were further obtained from a diversity of oral soft tissue, including the incisive papillae and the rugae area of the palate [16], the maxillary tuberosity [17], the oral mucosa [18], and the hyperplastic gingiva [19].

## 2. Effects of Microenvironment on Oral MSCs

### 2.1. Oral MSC Behavior under Inflammatory Microenvironment

The oral cavity is a distinctive habitat, harbouring an array of microorganism, with more than 700 species [20, 21]. An understanding of the effect of the resultant bacterially induced inflammatory environment on MSCs and their intrinsic processes is pivotal, in order to boost their reparative/regenerative potential and maintain their stemness. MSCs' survival under intensely inflamed microenvironmental conditions is relatively short, with a half-life of 24 hours [22], and the MSCs number is quite sparse [23, 24]. Three days following SHED injection into periodontitis-induced defects, minimal cell diffusion was detected with bioluminescence imaging, and SHED diminished during days 5 to 7 [24]. Similarly, three days following local or systemic injection of 1 × 10^7^ BM-MSCs into an injured corneas, surprisingly, less than 10 BM-MSCs appeared in the corneas, with a partial reversal of the deleterious effects of corneal opacity and inflammation [23]. This finding is completely opposite to the old concept that significant numbers of MSCs are required in injured sites for tissue repair. The theory of “hit-and-run” was proposed [25] to describe this rapid action of MSCs, including the secretion of soluble factors or secretomes, including cytokines, soluble factors, peptides, proteins, metabolites, microRNAs, and even mitochondria in extracellular vesicles [26–30].

Multiple investigations exploring the impact of the inflammatory microenvironment on oral MSCs' properties addressed their proliferation potential, migration and homing, multilineage differentiation, and inflammatory cytokines production [3]. Yet, results remain quite controversial, depending on the origins of MSCs under investigation, the variation and concentration of the inflammatory stimuli, and the experimental setup [31–33]. Regarding MSCs' proliferation, *Escherichia coli* lipopolysaccharide (*Ec*-LPS) inhibited the proliferation of DPSCs significantly at a concentration of 10 *μ*g/ml, while promoted it at 0.1 *μ*g/ml and exerted no influence at the tipping concentration of 1 *μ*g/ml [32], in a dose-dependent pattern. *Ec*-LPS enhanced the proliferation rate of BM-MSCs at 1 *μ*g/ml but not at 10 *μ*g/ml [34]. Also, BM-MSCs' attributes were demonstrated to be affected by different *Pg-*LPS concentrations, with an increase in proliferation, osteogenic differentiation, and immunomodulatory properties observed at 0.1 *μ*g/ml but a decrease or even apoptosis occurring at 10 *μ*g/ml [33]. Interestingly, compared to GMSCs from healthy tissues, the proliferation rate of GMSCs from inflamed tissues was enhanced [35]. Contrastingly, the proliferation of DFSCs was not influenced by concentrations of *Pg*-LPS ranging from 1 *μ*g/ml to 50 *μ*g/ml [36]. It appears that the source of oral MSCs could greatly affect their proliferative response to inflammatory stimuli of various concentrations.

Cellular proliferation is classically followed by selective migration/homing of MSCs in tissue regenerative/reparative process. This cellular homing and migration ability is principally regulated by local cytokines and chemokines [37]. PDLSCs cultured in interleukin-1*β* (IL-1*β*) (5 ng/ml)/tumor necrosis factor- (TNF-) *α* (10 ng/mL) inflammatory microenvironment upregulated their expression of CXC chemokine receptor 4 (CXCR4), which downregulated the tissue-released chemokine SDF-1/CXCL12 by bonding to it, thereby increasing PDLSCs' homing activity [38]. Moreover, the proinflammatory chemokine RANTES/CCL5 increased the migrated activity of PDLSCs, through the enhancement of the actin skeleton, the focal adhesion, the corresponding ECM receptors, and the cellular migration signaling pathway [39]. In conclusion, a controlled inflammatory microenvironment could significantly affect the homing and migration ability of oral MSCs.

Interestingly, LPS-challenged oral MSCs altered their regenerative markers' expression profile according to the source of the LPS. After stimulated by *Ec*-LPS, adipose stem/progenitor cells, DPSCs, and PDLSCs increased their osteogenic mRNA expressions, including alkaline phosphatase (ALP), Runt-related transcription factor 2 (RUNX2), osteocalcin (OCN) [32, 40, 41]. BM-MSCs, and adipose stem/progenitor cells also upregulated the calcium deposit as well as ALP activity with the stimulation of LPS (origin unclear) [31, 42]. In contrast, the expression of Col-I and OCN in human PDLSCs was downregulated upon *Pg*-LPS stimulation [43] and the calcium deposition by mouse BM-MSCs decreased by challenging them through *Ec-*LPS at 1 *μ*g/ml [44]. With the stimulation of IL-1*β* and TNF-*α*, GMSCs' osteogenic and adipogenic differentiation was suppressed [45]. Remarkably, GMSCs isolated from human hyperplastic gingival tissue displayed the same immunoregulatory functions in murine skin allograft as that of GMSCs from healthy gingiva, but weaker capability of collagen regeneration [19].

On the level of inflammatory cytokine release, a short-termed *Ec-*LPS stimulation of BM-MSCs, umbilical cord stem/progenitor cells, DFSCs, and DPSCs demonstrated a marked increase in their IL-6 and IL-8, but not in TNF-*α* secretion [46–48]. A long-term stimulation of PDLSCs by *Pg*-LPS resulted in a significant increase in cellular IL-1*β*, IL-6, and IL-8 release [43]. Compared to GMSCs from healthy tissue, GMSCs from inflamed tissue significantly produced higher IL-1, IL-6, and TNF-*α* [35]. In contrast, DFSCs challenged by *Pg*-LPS demonstrated no difference in IL-6 expression [36, 48].

Particularly, our group has been addressed in the study of GMSC behavior under inflammatory environment for years and uncovered that they were a unique group of MSCs with the characteristic of inflammatory resistance. Stimulation of GMSCs with *Pg*-LPS in concentrations ranging from 10 ng/ml to 10 *μ*g/ml upregulated their proliferation while induced minimally their inflammatory response and did not attenuate their regenerative capacity [49]. Challenged by a proinflammatory cytokines “cocktail” (IL-1*β*, TNF-*α*, and IFN-*γ*), the cellular proliferation of GMSCs declined at the initial stage (day 3) but increased significantly at the later stage (day 7), in a time-dependent manner. Their stemness and multilineage differentiation potential kept quite stable under “cocktail” stimulation [50].

Toll-like receptors (TLRs) expressed on MSCs were demonstrated to not only affect their migration, proliferation, and differentiation potential but also to play an important role in interaction with inflammatory environment. Therefore, we depicted TLR expression profile of GMSCs, DPSCs, and BM-MSCs under uninflamed [51–53] and inflamed conditions [51, 52]. In a basic medium, DPSCs expressed TLRs 1–10 in different quantities, while in inflammatory medium TLRs 2, 3, 4, 5, and 8 were upregulated, TLRs 1, 7, 9, and 10 downregulated, and TLR6 expression abolished [52]. Similarly, in a basic medium, GMSCs expressed TLRs 1, 2, 3, 4, 5, 6, 7, and 10, while in an inflammatory medium TLRs 1, 2, 4, 5, 7, and 10 significantly upregulated and TLR 6 diminished [51]. These variations in the TLR expression profile affect the recognition ability of MSCs for both gram-positive as well as gram-negative pathogens as well as damage-associated molecular patterns under inflammation [54, 55].

### 2.2. Oral MSC Behavior under Other Microenvironments

Oxidative stress, defined as the damage caused by an imbalance between oxidants and antioxidants [2], is two sided: high oxidant levels can damage biomolecules, while low basal level of oxidant is essential to the life processes [3]. Oxidative stress is tightly related to inflammation, affecting the development and perpetuation of inflammation in different stages and at various degrees [1]. Besides, reactive oxygen species (ROS), generated from mitochondrial complexes I and III and from NADPH oxidase isoform NOX4 during MSCs differentiation [4], could inhibit osteogenic differentiation [56] and induce adipogenesis/chondrogenesis [57, 58] if ROS level is excessive. BM-MSCs isolated from populations with old age, atherosclerosis, and type 2 diabetes, who experienced high oxidative stress, failed to induce T cell suppression [59, 60]. Nevertheless, the evidence of oxidative stress on oral MSCs is still quite rare and the intrinsic signaling is unclear.

Extracellular matrix (ECM) is also considered a crucial factor to influences oral MSCs by changing its biochemical or physical properties. Compared to collagen type I which is the most commonly purified ECM, decellularized bone extracellular matrix (bECM) hydrogels facilitated a significant upregulation of RUNX-2 and bone sialoprotein (BSP) of DPSCs, indicating their osteogenic differentiation [61]. Physical factors of ECM, such as stiffness, could influence the distribution and morphology of DPSCs and promote their odontogenic and osteogenic differentiation [62].

Micro-/nanoparticles have been widely used as bone graft substitute to mimic natural bone tissue. The addition of nanosilicates to poly(glycerol sebacate) could not only modulate the degradation rate and mechanical stiffness of the scaffold but also enhance the adhesion, spreading, proliferation, and osteogenic differentiation potential of preosteoblasts [63]. It was reported that compared to traditional biphasic calcium phosphate (BCP), BCP bioceramics composed of microwhiskers and nanoparticles hybrid-structured surface (hBCP) employed in a long bone defect model of beagle dogs achieved a higher quality of regenerated bone and a higher fracture load. Also, hBCP group dramatically downregulated inflammatory gene expression of BM-MSCs, indicating a closer resemblance of hBCP to the natural bone [64].

## 3. Effects of Oral MSCs on Inflammatory Microenvironment

### 3.1. “Double-Edged Sword” Effect of Oral MSCs on Their Inflammatory Microenvironment

Various experimental setups including animal models, cellular coculture system, and conditioned medium application were administrated to explore the impact of MSCs on inflammatory microenvironment, including immune cell infiltration and inflammatory cytokine production. The immunomodulatory effects of oral MSCs are believed to be mediated by direct cell to cell contact as well as through the production of soluble cytokines, including IL-1, IL-6, IL-10, indoleamine 2,3-dioxygenase (IDO), nitric oxide (NO), transforming growth factor (TGF)-*β*1, and prostaglandin E2 (PGE2) [65].

**Figure 2:**
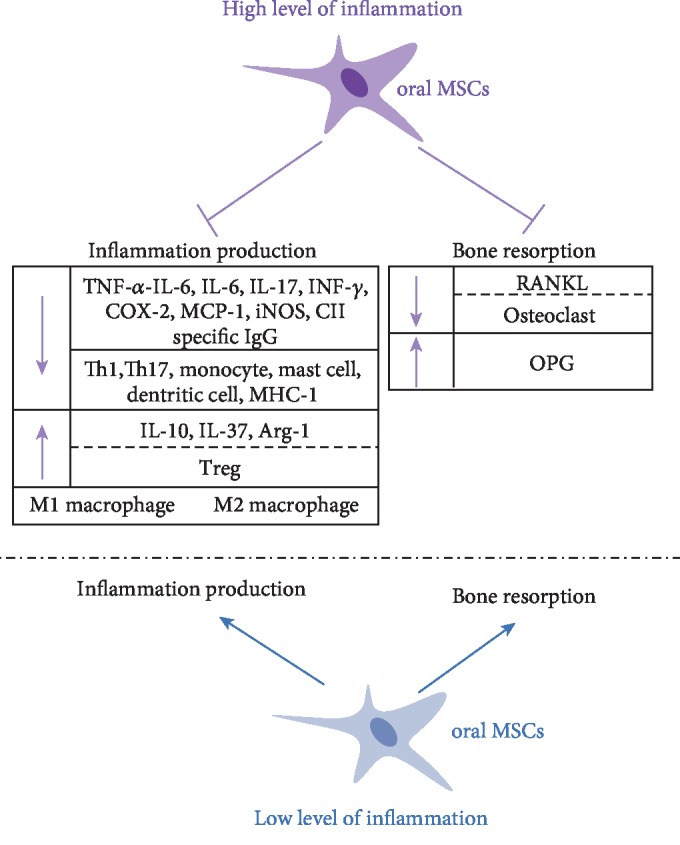
Under a high level of inflammation, oral MSCs suppress inflammation production and bone resorption by the regulation of inflammatory cytokines, inflammatory cells, RANKL/OPG, and osteoclasts, respectively. While under a low level of inflammation, oral MSCs promote both inflammation production and bone resorption.

Yet, the immunoregulative capacity of MSCs is largely governed by the surrounding inflammatory intensity [5]. Under low inflammatory condition, MSCs promote the inflammatory response through the secretion of cytokines that recruit immune cells to the local area, while if the inflammatory cytokines exceed a certain threshold, MSCs shift from pro- to anti-inflammatory cells, preventing an overexpression of immunoreaction [5, 66] (Figure 2). MSCs exert an inhibitory effect on effector T cells under high concentrations (0.4 ng/ml) of interferon- (IFN-) *γ* and TNF-*α*, while under low concentrations (0.2 ng/ml) of the same cytokines, MSCs promote their cellular proliferation. This “double-edged sword” effect is attributed to the inflammatory-dose-dependent production of NO/IDO by MSCs, which functions as an “on-off” switch, changing MSCs from being immunosuppressive to immune enhancing [66]. Similarly, a low dose of IFN-*γ* could boost the antigen-presenting functions of MSCs through upregulating MHC-II and MHC-I, rendering them less susceptible to NK-mediated cell lysis. High doses of IFN-*γ*, contrastingly suppress the expression of the same MHCs in MSCs [67, 68].

In the periodontium, the inflammatory concentration is directly associated with the severity of periodontitis [69]. PDLSCs clearly demonstrated similar “double-edged sword” properties. In a healthy periodontium, PDLSCs suppress the production of ROS by neutrophil precursor HL-60D, exerting a protective effect on the surrounding tissues from ROS-mediated deleterious attack. Yet, with a challenge of *Porphyromonas gingivalis* total protein extract (*Pg*-PE), PDLSCs reverse their action, augmenting the ROS production by activating HL-60D [70–74].

Similarly, the effects of MSCs on osteoclast also depends on the dose of the surrounding biomolecules as well as the concentration of the inflammatory condition [75]. Osteoclast formation was enhanced when cocultured with BM-MSCs in the presence of 10^−9^ M 1*α*,25(OH)2D3. At higher concentrations of 10^−8^ M 1*α*,25(OH)2D3, this effect was not observed [76]. Similarly, BM-MSCs promoted the formation and function of cocultured osteoclasts in the absence or presence of a low dose of TNF-*α* (5 ng/ml), while inhibited osteoclast formation at high doses of TNF-*α* (10 ng/ml) [77]. Receptor activator of NF-*κ*B ligand (RANKL) is expressed by MSCs during normal physiological remodeling, while under inflammatory conditions the expression of OPG [78] and IL-10 [79] is upregulated and RANKL [77] is slightly downregulated. This dual regulatory effect of MSCs on the formation and differentiation of osteoclasts is further consistent with the “double-edged sword” property of MSCs in inflammatory regulation, switching between proinflammatory and anti-inflammatory phenotypes by sensing different inflammatory milieu (Figure 2). Thus, aside from playing a classical role to ameliorate inflammation, MSCs could serve as inflammatory promoters to activate immune cells under certain conditions. Through such a dose-dependent feedback, MSCs maintain tissue integrity and homeostasis as an immunoregulative agent [5, 80].

As above, MSCs and the local inflammatory environment could affect each other under the inflammatory stimuli. Although they seem to be two independent biological behavior, underlying correlations exist to maintain tissue equilibrium. The influence of inflammatory environment on MSCs would affect their survival, proliferation, stemness, and differentiation potential, which might impair or boost the process of tissue regeneration/repair. Also, MSCs' actions as immunomodulators could attenuate the inflammatory process.

### 3.2. Effects of Oral MSCs on the Innate and Acquired Immune System

**Figure 3:**
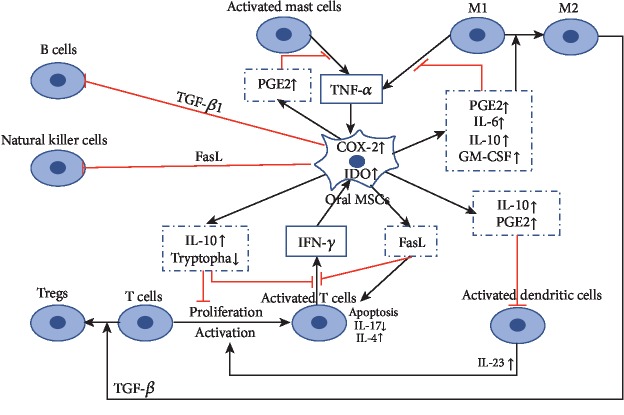
Immunomodulatory “crosstalk” between oral MSCs and mast cells, macrophages (with their M1 and M2 phenotypes), dendritic cells, natural killer cells, T cells, and B cells. COX-2: cyclooxygenase-2; GM-CSF: granulocyte-macrophage colony-stimulating factor; IDO: indoleamine 2,3-dioxygenase; IFN: interferon; IL: interleukin; PGE2: prostaglandin E2; TGF: transforming growth factor. Red lines: inhibition, black arrows: secretion/stimulation, blue dotted line box: soluble factors and transmembrane protein secreted by oral MSCs, blue solid line box: stimuli secreted by immune cells.

Oral MSCs regulate local inflammatory environment also by interacting with the innate and acquired immune system in a multifaceted way (Figure 3). As primary antigen-presenting cells, dendritic cells (DCs) couple innate and adaptive immune response [81]. Both DPSCs and GMSCs have been demonstrated to interfere with the maturation and activity of DCs, lessening their antigen presentation competence and inflammatory reaction, which may attribute to the elevation/activation of IL-10 via a PGE2-mediated mechanism [82, 83]. Moreover, by interacting with EP4 receptors (PGE2-receptor subtype) expressed on DCs, PGE2 secreted from GMSCs increases the production of IL-23 in DCs, which facilitates Th17 cell expansion [84].

Macrophages, which are indispensable components of the innate immune system, contain two major subpopulations, namely proinflammatory M1 phenotype and anti-inflammatory M2 phenotype [81]. DPSCs are reported to inhibit M1 macrophage function by preventing their TNF-*α* secretion via an IDO-mediated pathway [85]. Also, DPSCs as well as GMSCs possess the ability to guide the polarization of macrophages toward M2 phenotype, through elevated secretion of PGE2, IL-6, IL-10, and GM-CSF [86, 87].

Mast cells play a significant role in allergy and inflammation. Studies show that GMSCs hinder the production of TNF-*α* in activated mast cells, which is believed to be partly mediated by TNF-*α*/PGE2 feedback axis [83, 86].

Lymphocytes, consisting of T cells, B cells, and natural killer cells (NK cells), play a vital role in the adaptive immune response. GMSCs were demonstrated to inhibit the proliferation/activation of T cells in vitro through upregulating IL-10 and downregulating tryptophan, via GMSCs' derived indoleamine 2,3-dioxygenase (IDO) [86, 87]. IFN-*γ* released by activated T cells acts as a regulator in the feedback signaling between T cells and GMSCs [87]. Additionally, GMSCs, DPSCs, PDLSCs, and SHED possess the capability to suppress activation of Th17 cells [88–91] and promote the growth of CD4^+^CD25^+^FoxP3^+^ regulatory T cells (Tregs) [88–90], mediated by M2 macrophages via a TGF-*β*-dependent mechanism [88, 89]. Studies also revealed that GMSCs and DPSCs induced T cell apoptosis and suppressed the proliferation of NK cell and Th1 via FasL/Fas-mediated pathway [92, 93], resulting in the decreased production of IFN-*γ* and IL-17 by Th1, whereas it enhanced IL-4 secretion by Th2 cells. As to the humoral immunity level, similar to other MSCs, PDLSCs and DPSCs were considered to hinder B cell proliferation, differentiation potential, and antibody production [88, 94] and to suppress allogeneic T and B cell proliferation through TGF-*β*1 release [88].

Moreover, GMSCs, SCAPs, PDLSCs, DFSCs, and DPSCs are capable of suppressing the proliferation and activation of human peripheral blood mononuclear cells (PBMCs). GMSCs, PDLSCs, and SCAPs were reported to exert an adverse effect on PBMCs [17, 87, 95, 96], in a dose-dependent manner [17, 87, 95], through their secretion of TGF-*β*, HGF, and IDO [96]. Similarly, DPSCs and DFSCs hindered the proliferation of PBMCs by producing TGF-*β* [96, 97] and TLR4 agonists could facilitate this effect [97].

## 4. Oral MSC-Derived Extracellular Vesicles (EVs) in Conditioned Media

To date, the intrinsic immunoregulative mechanism of MSCs are not fully elucidated, although paracrine factors have been corroborated to play an important role in this process. The paracrine action may be attributed, at least in part, to EVs released from MSCs in their respective media. EV, known as nanosized membrane structures released by cells in an evolutionally conserved manner, can transfer RNA, microRNA, and even proteins to modulate inflammatory environment [98–100]. EV can be broadly divided into four main categories: exosomes, microvesicles, retrovirus-like vesicles, and apoptotic bodies. Exosomes, arising from exocytosis of multivesicular bodies with the size of 30-120 nm, are encapsulated within a lipoprotein coat, which determines its tropism and protects it from being degraded in the systemic circulation. Microvesicles, budding from the plasma membrane, are vesicles at 50–2,000 nm. Retrovirus-like particles are noninfectious vesicles at 90–100 nm that are similar to retroviral vesicles and contain a portion of retroviral proteins. Apoptotic bodies are vesicles at 50–5,000 nm that are produced from cell death during apoptosis progress [101–103].

Oral MSC-derived exosomes have been reported of therapeutic benefit in a number of inflammatory-related disease, through different molecular mechanisms. BM-MSC-derived exosomes promoted the regeneration/repair of the periodontal ligament and temporomandibular joint, through activation of AKT, ERK, and AMPK signaling pathways that suppress the inflammatory response by chondrocyte and increase the proliferation and migration of periodontal ligament cells [104, 105]. Following stimulation by *Pg*-LPS, microRNA-155-5p expression in PDLSC-derived exosomes significantly decreased and the exosome was ingested by CD4^+^ T cells. MicroRNA-155-5p could negatively regulate its downstream target molecule in CD4^+^ T cells, known as Sirtuin-1. Therefore, the downregulation of microRNA-155-5p in PDLSC-derived exosomes by *Pg*-LPS rescued the Th17/Treg imbalance via upregulating Sirtuin-1 in CD4^+^ T cells [106].

The same exosomes were also demonstrated to suppress the inflammatory cytokines in autoimmune encephalomyelitis, through inactivating NALP3 and NF-*κ*B pathways [98, 99]. DPSC-derived exosomes demonstrated the ability to reduce edema in carrageenan-induced acute inflammation model, with an effect comparable to prednisolone. Still, prednisolone exerted a prominent inflammatory suppression at early stages, while DPSC-derived exosomes inhibited the inflammation more reliably at later time points [107]. SHED-derived exosomes were effective in treating traumatic brain injury by promoting motor functional recovery, reducing cortical lesion, and decreasing neuroinflammation at cellular levels, through shifting microglia from M1 pro- to M2 anti-inflammatory phenotype [108]. In an acute lung injury mouse model, a single intravenous administration of SHED-secreted factors alleviated lung injury, weight loss, and inflammatory response, accompanied by an increased number of anti-inflammatory M2-like lung macrophages [100]. GMSC-derived exosomes were capable of accelerating wound healing in diabetic mice by promoting reepithelialization, angiogenesis and neuronal ingrowth, and collagen remodeling [109]. Particularly, exosomes derived from healthy BM-MSCs alleviated radiation-induced bone loss by recovering the adipogenic and osteogenic differentiation potential of radiation-injured BM-MSCs via Wnt/*β*-catenin activation [110].

Additionally, BM-MSC-derived microvesicles downregulated the level of IL-1*β*, IL-6, TNF-*α*, iNOS, and PTGS2, produced by microglia cells, when cocultured in LPS inflammatory environment. Moreover, the phosphorylation of ERK, JNK, and p38 molecules in microglia cells was also suppressed by BM-MSC-derived microvesicles [111]. In an uninflamed environment, DPSCs were shown to induce the production of TNF-*α* by macrophages via PKR-rich microvesicles [112].

Although EVs in the oral MSC-derived conditioned media play a pivotal role in cell-cell communication, the information exchange between oral MSCs and their surrounding inflammatory microenvironment is bidirectional. EVs liberated from oral MSCs could restrain the inflammatory response in immune cells, and conversely immune cells could affect the proliferation, differentiation, and migration potential of MSCs, through their EVs [113, 114]. In an *Ec*-LPS-challenged inflammatory environment, monocyte-derived exosomes could promote the osteogenic differentiation of BM-MSCs [115]. EVs derived from dendritic cells could increase the migration of BM-MSCs in a dose-dependent manner, even in the absence of dendritic cells [116].

In this context, EV-based approaches appear to provide a new paradigm for cell-free therapies, overcoming many of the current clinical constrain of cellular transplantation. EV, encapsulating prolific proteins and RNAs, can cross the plasma membrane to deliver their cargo into target cells and are tolerated by the body [55]. Moreover, it was suggested that exosome secretion profile can be improved by preconditioning or genetic modification of the parent cells, so it could be an ideal vehicle for drug and gene delivery [117, 118]. On the other hand, contents of MSCs' EVs are not always static but vary according to the surrounding environment and in different growth periods of MSCs. Therefore, we still scarcely recognize the proteomic and genomic complexities of EVs and further studies are needed to investigate the exact composition and possible mechanism.

## 5. Experimental Therapeutic Applications of Oral MSCs

### 5.1. Experimental Therapeutic Applications of Oral MSCs in Periodontal Diseases

Compared to other MSCs, oral tissue-derived MSCs are more adaptive to inflammatory environmental challenges, principally because of the bacteria-congregated habitat they usually reside in. The complex subgingival microenvironment is an ideal cradle for bacteria colonization, where it inhabits an array of diverse bacterial species known as subgingival plaque [119]. Still, despite the huge number of microorganisms, the subgingival community is quite stable and harmonious. The occurrence of an environmental imbalance results in the initiation of a periodontal disease with a marked bacterial dysbiosis, the multiplication of various periodontal pathogens, and a shift in the bacterial ecology from gram-positive aerobic to gram-negative anaerobic form [120].

Under these pathogenic conditions, oral MSCs could exert a variety of anti-inflammatory functions (Table 1). In inflammatory periodontitis-induced bone defect, MSCs were demonstrated to mediate the formation of new bone- and periodontal ligament fiber-like structures [121], through decreasing the number of TRAP^+^ (a specific histochemical marker of osteoclasts) cells and attenuating the level of the proinflammatory cytokines TNF-*α* and IL-17, as well as increasing the anti-inflammatory cytokine IL-10 and shifting M1 macrophage to a M2 phenotype [24, 122, 123]. SHED and BM-MSCs demonstrated an ability to mitigate the severity of periodontal bone loss, with a decrease in the concentrations of TNF-*α*, IL-1*α*, IL-1*β*, IL-17, and IFN-*γ* and an increase in IL-10 [24, 122–124]. Pretreatment of BM-MSCs by acetylsalicylic acid could further augment these effects [124]. SHED significantly amplify the number of CD206^+^ (M2) macrophages in periodontal tissues. It further promote the conversion of M1 macrophages which secrete NO, ROS, and TNF-*α* to CD206^+^ M2 macrophages which release IL-10, TGF-*β*, and arginase-1 (Arg1), in a coculture system [24, 125]. Similarly, BM-MSC-conditioned medium downregulated mRNA expression of IL-1, IL-6, and TNF-*α*, as well as the IL-6/IL-10 ratio in a rat tooth transplantation model [126]. BM-MSCs also promoted bone-related factor expression by alveolar osteoblasts (AOs), gingival fibroblasts (GFs), and periodontal ligament cells [127]. Additionally, BM-MSCs could reduce alveolar bone loss, osteoclast number, inflammatory cell infiltration, and RANKL/OPG ratio in periodontitis-induced bone defect [123, 124]. In orthodontic movement, BM-MSC administration, especially BM-MSCs transfected with OPG plasmids, significantly decreased orthodontic force-induced root resorption lacunae, osteoclast number, RANKL, and COX-2 [128].

Thus, through their regenerative and paracrine immunomodulatory actions, oral MSCs augment periodontal tissue regeneration, while ameliorating bone resorption. They decrease inflammatory factor level and RANKL/OPG ratio in periodontal disease by regulating immune cells, resident cells, and osteoclasts, so as to mitigate bone loss. This immunoregulative function and inflammatory resistance in turn maintain their stemness, survival, and multilineage differentiation potential, in order to promote periodontal tissue regeneration/repair.

### 5.2. Experimental Therapeutic Applications of Oral MSCs in Systemic Diseases

Oral MSCs are applicable not only in oral disease but also in systemic diseases as immunoregulators, mainly through decreasing inflammatory cytokine production, inhibiting bone resorption in a cell-cell contact manner or through a paracrine way [65] (Table 1).

Acute lung injury is characterized by an excessive and uncontrolled inflammatory response, which involves the activation of neutrophils and macrophages and the corresponding tissue damage by releasing antimicrobial compounds [129]. Tail intravenous introduction of DFSC/DFSC-conditioned medium into an acute lung injury model alleviated the histopathological damage and pulmonary permeability through downregulating MCP-1, IL-1*β*, IL-6, and TNF-*α*, as well as upregulating the levels of IL-10 and the macrophage M2 marker Arg-1 in bronchoalveolar lavage fluid [129]. Also, the compromised alveolar epithelial cell properties including the decreased viability, increased apoptosis, increased NF-*κ*B activity, and extrainflammatory cytokine release were reversed by BM-MSC administration [130].

Multiple sclerosis is a chronic inflammatory, demyelinating disease of multiple pathogenic factors, which is characterized by myelin degradation and damage of the central nervous system. PDLSCs were corroborated to mitigate the inflammatory response by inhibiting TNF-*α*, COX-2, and iNOS expressions and concomitantly enhancing IL-10 and IL-37 secretion in the experimental autoimmune encephalomyelitis, which is a murine model of multiple sclerosis. Furthermore, PDLSCs were demonstrated to suppress cellular apoptosis by inhibiting the proapoptotic protein caspase-3 and enhancing the antiapoptotic protein Bax and Bcl-2 [131].

Optic neuropathies, the leading cause of irreversible blindness and visual impairment, which result from trauma injury, glaucoma, inflammation, ischemia, or tumor compression, are characterized by the degeneration of retinal ganglion cells (RGCs) and their axons. Intravitreal injection of PDLSCs into the rat vitreous chamber in the optic nerve injury model could promote the survival and neurite regeneration of RGCs by increasing brain-derived neurotrophic factor secretion in a direct cell-cell-contact manner [132].

Sjögren's syndrome is a systemic autoimmune disease that primarily affects the exocrine glands. It is characterized by clinical symptoms including dry eyes and mouth, and histological feature of focal lymphocytic infiltration of the exocrine glands. Currently, the role of CD4^+^ T cells in the pathogenesis of Sjögren's syndrome has been proposed, and multiple studies have demonstrated the role of MSCs in the regulation of T cells. In the murine Sjögren's-like disease model, BM-MSCs restored the salivary flow rate and reduced the lymphocytic infiltration by modulating pro- and anti-inflammatory cytokines INF-*γ*, TNF-*α*, IL-10, PGE2, and IL-6 [133].

Type 1 diabetes is an autoimmune disease caused by the immune-mediated destruction of pancreatic *β*-cells, involving the upregulation of inflammatory cytokines and the imbalance of Th17 cells and Tregs. GMSCs were demonstrated effective in the treatment for type 1 diabetes, with the better control of blood glucose levels, the delayed diabetes onset, and the ameliorated pathology scores in the pancreas. The mechanism was postulated to be primarily mediated through downregulating the level of IL-17 and IFN-*γ* expressed by CD4^+^ and CD8^+^ T cells and upregulating the number and function of Tregs [134]. Moreover, diabetic polyneuropathy, the most common microvascular complication of both type 1 and type 2 diabetes, involves inflammatory response during the disease development. DPSC introduction ameliorated diabetic polyneuropathy, improving the delay in sciatic nerve conduction velocities and the decreased nerve blood flow, through downregulating monocytes/macrophages, inflammatory messenger ribonucleic acid and upregulating CD206 mRNA (a M2 macrophage marker) [135].

Allergic contact dermatitis, classified as a type IV hypersensitivity reaction, is caused by repeated skin exposure to contact allergens. Contact hypersensitivity (CHS) is a classic murine model of allergic contact dermatitis, in which dermal DCs, allergen-specific T lymphocytes, allergen-specific effector T cells, and mast cells play a significant role. Infusion of GMSCs into the CHS reversed the imbalance of abnormal Th1/Th2 ratio and decreased the infiltration of dendritic cells, mast cells, and Th17. It further increased the number of Tregs in the allergen contact area, with a remission of hypersensitivity. Mechanically, GMSCs inhibited proinflammatory factors and promoted anti-inflammatory factors through COX/PGE2 and PGE2–EP3 signaling pathways [83, 136]. Interestingly, in the CHS model, a local injection of GMSCs demonstrated a more marked effect on attenuating inflammation than intravenous injection of the same cells [83, 136]. An intravenous injection of GMSCs improved arthritis, with the reduced TNF-*α* and CII-specific IgG secretion via FasL/Fas pathway [137]. Similarly, allergic rhinitis, which is an allergic disease defined as inhaled-particle-induced nasal mucosa hypersensitivity, can trigger an IgE-mediated hypersensitivity. SHED administration significantly reduced nasal symptoms and inflammatory infiltration in allergic rhinitis [138].

Colitis is an inflammatory-related colonic mucosal injury in the distal small intestine, contributing to the dysfunction of innate and adaptive immunity. GMSCs and DPSCs significantly ameliorated inflammation clinically and histopathologically, by restoring the normal intestinal architecture, reversing weight loss, improving the overall disease score, and suppressing epithelial ulceration. At the cellular and molecular level, GMSCs and DPSCs decreased infiltration of Th1 and Th17 cells, increased Tregs, inhibited IL-6 and IL-17 release, and elevated IL-10 level [87, 93].

Rheumatoid arthritis, a multisystem autoimmune disease characterized by the loss of immunologic self-tolerance and chronic inflammation, could impair the joints of the whole body. GMSCs was reported to mitigate bone and cartilage destruction by remarkably enhanced Tregs number through CD39/CD73 signals in the murine-rheumatoid-arthritis model [139].

Process of cutaneous wound healing can be divided into three stages: inflammation, tissue formation, and remodeling, which involves interactions of resident cells, infiltrating immune cells, and their secretion as well. GMSCs were found to interact with the local inflammatory microenvironment, migrating into the local wound bed and promoting macrophage conversion from M1 to M2 phenotype. Concomitantly, TNF-*α* and IL-6 were suppressed while IL-10 was elevated with the administration of GMSCs, with accelerated wound healing, rapid deepithelialization, and increased angiogenesis [140].

Moreover, spinal cord injury (SCI) normally caused by accident and inflammation is considered a key regulator during the secondary neurodegenerative events [141]. SCAP advanced the healing of spinal cord injury through reducing TNF-*α* level and promoting the differentiation oligodendrocyte progenitor cells. Besides, SHEDs/SHED-conditioned medium were found to promote the fibrotic scar resolution, by suppressing the inflammatory mediators TNF-*α*, IL-1*β*, and iNOS and by inducing apoptosis of activated hepatic stellate cells, in the liver fibrosis experimental model [142].

As above, oral MSCs contributed to tissue regeneration/repair in a variety of immune/inflammatory disease not only by boosting tissue regeneration but also by ameliorating immune/inflammatory response. However, the majority of studies still remained at the initial stage of observing the variation of inflammatory cytokines and immune cells or detecting the signaling pathway; exact mechanisms are needed to be further elucidated.

## 6. Clinical Applications of MSCs in Autoimmune and Inflammatory Diseases

Due to the immunomodulatory function and low immunogenicity of MSCs, autologous/allogenic MSCs obtain good results in the treatment of human autoimmune and inflammatory diseases. Clinical efficacy and safety have been demonstrated for MSCs applied in patients with systematic lupus erythematosus [143–145], rheumatic arthritis [146], GVHD [147], and osteoarthritis, [148] via intravenous or local injection, with the results of a decrease in the disease activity score, rebuilding of T cell imbalance, and functional improvement. Nevertheless, despite the inspiring outcomes in clinical trials, it was proposed that MSC transplantation might not be effective in refractory patients in a long-term perspective [149, 150].

As for oral MSCs, researchers discovered that GMSCs possess an extraordinary potential in the treatment of autoimmune and inflammatory diseases owing to their high proliferation rate, no tumorigenesis [151], immunomodulatory function, stable stem cellularity, and differentiation abilities under inflammatory environment [19, 152]. Moreover, GMSCs isolated from patients with rheumatic arthritis and systematic lupus erythematosus presented similar cell phenotype and immunosuppressive action [153]. Yet, clinical trials of GMSCs and other oral MSCs applied in patients with autoimmune and inflammatory diseases are still deficient, so further study is needed to reveal their immunomodulatory efficacy.

## 7. Conclusion

Oral MSCs, with the unique clinical advantages of easy access in large quantities, as well as their remarkable tissue reparative/regenerative potential, have been proposed as ideal candidates for MSC-based tissue regeneration. Moreover, the current knowledge reveals a vivid interaction between the oral MSCs and their inflammatory milieu, both at the cellular and secretomes levels. The interaction largely governs the proliferation potential, migration and homing, multilineage differentiation, and inflammatory response of oral MSCs on the one hand, and on the other hand, regulates the severity of local inflammatory microenvironment. Particularly, the bidirectional regulatory manner of MSCs, including anti-inflammatory and proinflammatory effects, boosts them to become novel natural modulators in maintaining inflammatory balance, not only in periodontitis but also in various systemic diseases. Nevertheless, we still scarcely know the underlying molecular mechanisms, though recently the concept of secretomes-driven cell-free therapeutic potential opens a multitude of very promising perspectives clinically. Thus, a deeper understanding of the underlying mechanisms of oral MSCs' immunomodulatory effects could pave the way to make MSC therapy a clinical reality.

## Figures and Tables

**Table 1:** The immunoregulatory effects of oral MSCs.

Type	Disease	Oral MSC	Function	Mechanism	References
Inflammatory disease	Periodontitis	BM-MSCs, SHEDs	Mitigate the severity of bone loss	Decrease TNF-*α*, IL-17, IFN-*γ*, IL-1*α*, IL-1*β*, osteoclasts, RANKL/OPG ratio, inflammatory cells; increase IL-10, M2-like macrophage	[24, 122–124]
	Acute lung injury	DFSCs	Alleviate histopathological damage and pulmonary permeability	Decrease MCP-1, IL-1*β*, IL-6, TNF-*α*; increase IL-10, Arg-1	[129]
	Colitis	DPSCs, GMSCs	Rescue weight loss, ameliorate colonic transmural inflammation, suppress epithelial ulceration, and restore normal intestinal architecture	Induce T cell apoptosis through Fas ligand decrease Th1, Th17 cells, IL-6, IL-17; increase Tregs, IL-10	[87, 93]

Autoimmune disease	Autoimmune encephalomyelitis	PDLSCs	Mitigate inflammatory response and apoptosis enhance IL-10 and IL-37 secretion, and suppress	Decrease TNF-*α* , COX-2, iNOS, caspase-3, Bax; increase IL-10, IL-37, Bcl-2	[131]
	Sjögren's-like disease	BM-MSCs	Rescue a decline in the salivary flow rate, reduce lymphocyte infiltration in the salivary gland	Modulate INF-*γ*, TNF-*α*, IL-10, PGE2, IL-6	[133]
	Rheumatoid arthritis	GMSCs	Inhibit inflammation, ameliorate bone and cartilage destruction	Increase Tregs; decrease TNF-*α*, CII-specific IgG; through FasL/Fas and CD39/CD73 signals	[137, 139]
	Type 1 diabetes	GMSCs	Decrease blood glucose levels, delay diabetes onset, ameliorate pathology scores in the pancreas	Decrease IL-17 and IFN-*γ*; increase the number and function of Tregs	[134]
	Diabetic polyneuropathy	DPSCs	Improve the delay in sciatic nerve conduction velocities and the decrease in nerve blood flow	Decrease monocytes/macrophages, ribonucleic acid; increase CD206 mRNA	[135]

Allergic disease	Allergic rhinitis	SHED	Reduce nasal symptoms and inflammatory infiltration	Decrease IL-4, IL-5, IL-13, IL-17A; increase IFN-*γ*	[138]
	Contact hypersensitivity (CHS)	GMSCs	Reduce hypersensitivity	Reverse the imbalance of abnormal Th1/Th2 ratio; decrease dendritic cells, mast cells, Th17; increase Tregs; PGE2-signaling pathway	[83, 136]

Others	Fibrotic scar	SHEDs	Promote fibrotic scar	Decrease TNF-*α* , IL-1*β*, iNOS; activate apoptosis of hepatic stellate cells	[142]
	Optic nerve injury	PDLSCs	Promote retinal ganglion cell survival and neurites regeneration	Direct cell-cell interaction, increase brain-derived neurotrophic secretion	[132]
	Bone resorption	BM-MSCs	Decrease orthodontic-force induced root-resorption lacunae	Decrease osteoclast number, RANKL, and COX-2	[128]

PDLSCs: periodontal ligament stem cells; DPSCs: dental pulp stem cells; SHED: stem cells from human exfoliated deciduous teeth; GMSCs: gingival mesenchymal stem cells; SCAP: stem cells of the apical papilla; DFSCs: dental follicle stem cells; BM-MSCs: bone marrow mesenchymal stem cell.
